# Supplementary material for: N-Acetyltransferase 10 represses Uqcr11 and Uqcrb independently of ac4C modification to promote heart regeneration
Source: Nat Commun. 2024 Mar 8;15:2137. doi: 10.1038/s41467-024-46458-7 (PMC10923914; doi:10.1038/s41467-024-46458-7)
Supplement: Supplementary file 1 — Supplementary information [file 41467_2024_46458_MOESM1_ESM.pdf]

N-Acetyltransferase 10 represses Uqcr11 and Uqcrb independently of ac4C modification to promote heart regeneration

Supplementary Figures

Supplementary Fig. 1

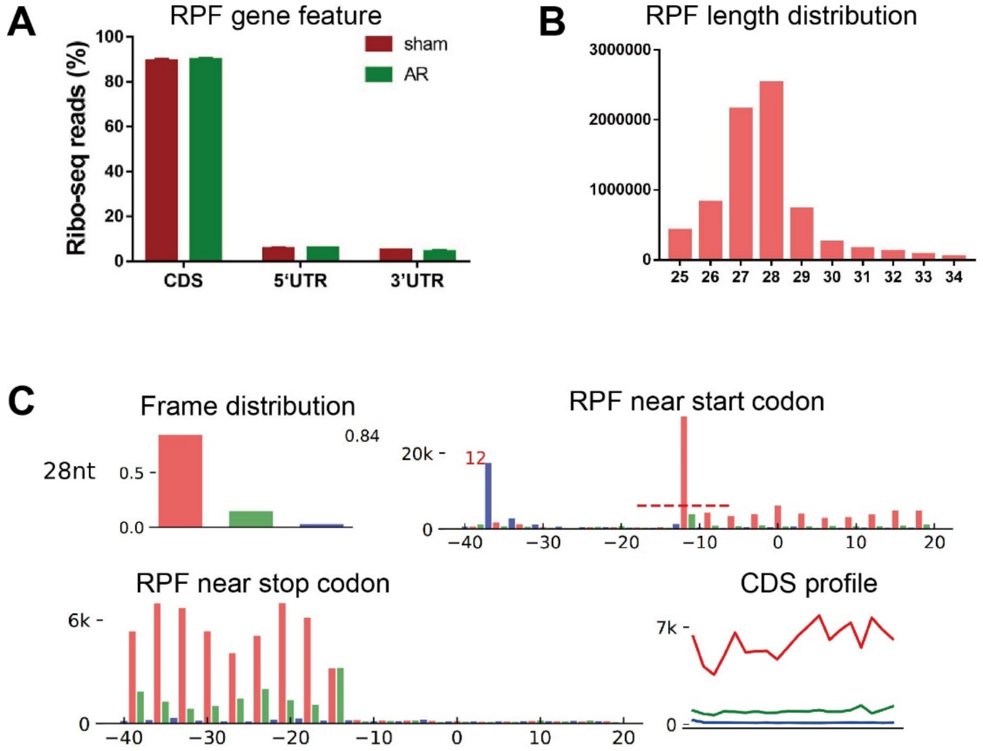

Supplementary Figure 1. The analysis of RPF. A The RPFs distributes in the CDS, 5'UTR and 3'UTR. **B** The RPF length distribution. **C** The ribosome footprint positions analysis shows the percentage of footprints that match these primary reading frames, the P-site positions, the 3-nt periodicity, and the CDS profile. Source data are provided as a Source Data file.

Supplementary Fig. 2

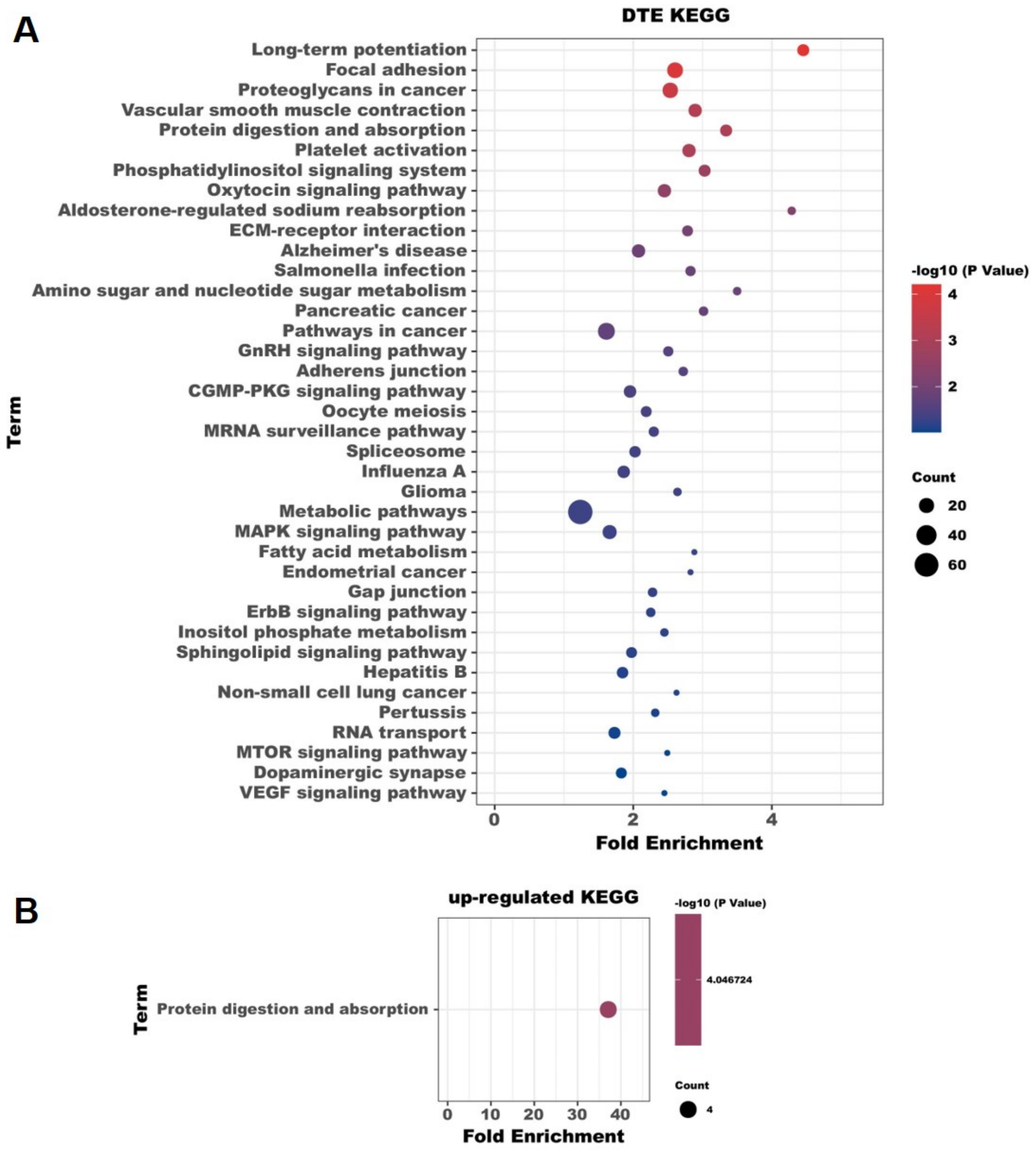

Supplementary Figure 2. The KEGG analysis. A KEGG analysis of differentially expressed TE. **B** KEGG analysis of genes with up-regulated translation by DAVID (https://david.ncifcrf.gov/home.jsp) and HIPLOT (https://hiplot.com.cn/cloud-tool/drawing-tool/list). Source data are provided as a Source Data file.

Supplementary Fig. 3

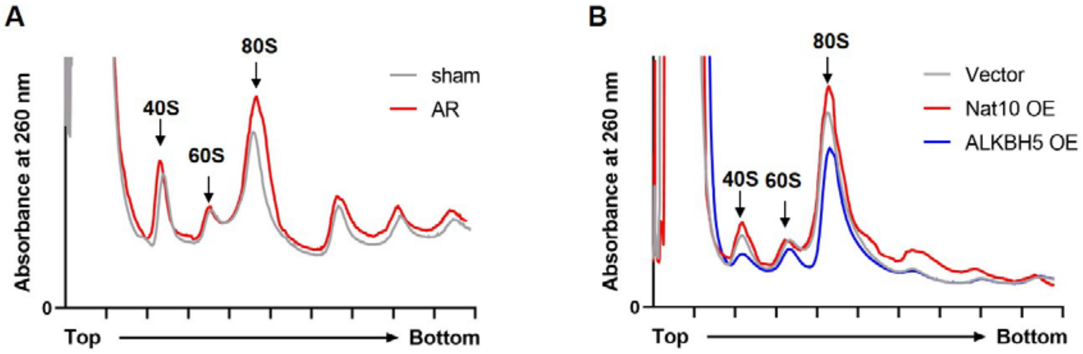

Supplementary Figure 3. A and **B** The A254 absorbance data of Polysome profiling analyses. Source data are provided as a Source Data file.

Supplementary Fig. 4

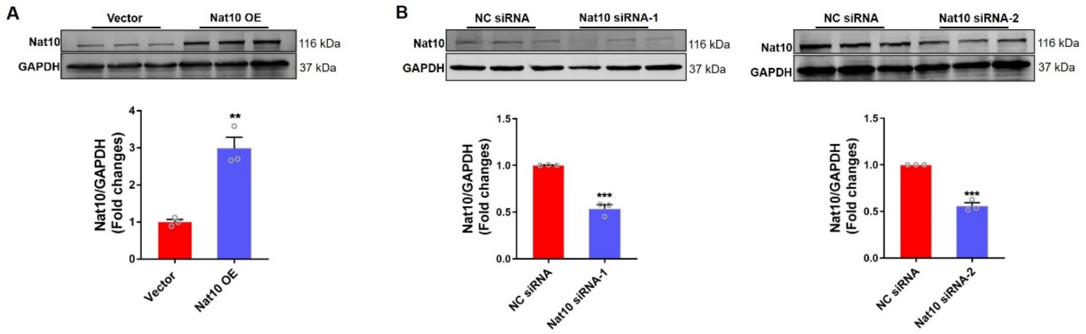

Supplementary Figure 4. The expression of Nat10 in cardiomyocytes transfected with Nat10 overexpression plasmid and siRNA. Source data are provided as a Source Data file. **A** Two-tailed Student’s t test. **p < 0.01 vs. Vector. **B** Two-tailed Student’s t test with Welch’s correction. ***p < 0.001 vs. NC siRNA. n=3. Data are presented as mean ± SEM.

Supplementary Fig. 5

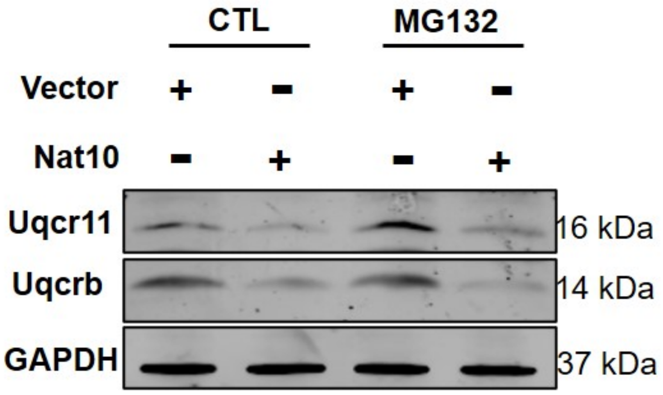

Supplementary Figure 5. The effect of Nat10 on Uqcr11 and Uqcrb protein degradation.

Supplementary Fig. 6

PACES Prediction of acetylation sites in mRNA

Result:

Gene	Sequence	Start	End	Score thresholds:
Uqcr11	no actylated region			null

Gene	Sequence	Start	End	Score thresholds:
Uqcrb	no actylated region			null

Gene	Sequence	Start	End	Score thresholds:
Atp5j2	no actylated region			null

Supplementary Figure 6. The prediction of ac4C modification. The ac4C modification sites in Uqcr11, Uqcrb, and Atp5j2 mRNAs were predicted with 99% specificity by PACES (http://www.rnanut.net/paces/).

Supplementary Fig. 7

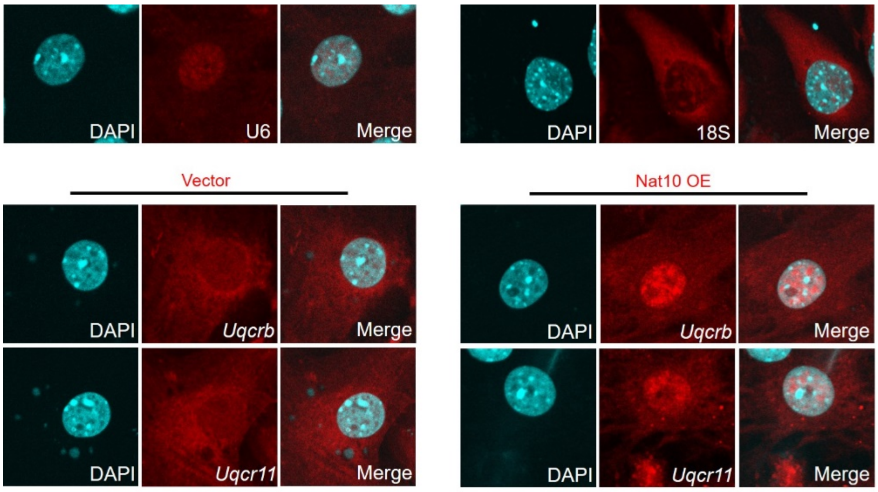

Supplementary Figure 7. The distribution of *Uqcr11* and *Uqcrb* between the nucleus and cytoplasm. The RNA localization of Uqcr11 and Uqcrb assessed using FISH.

Supplementary Fig. 8

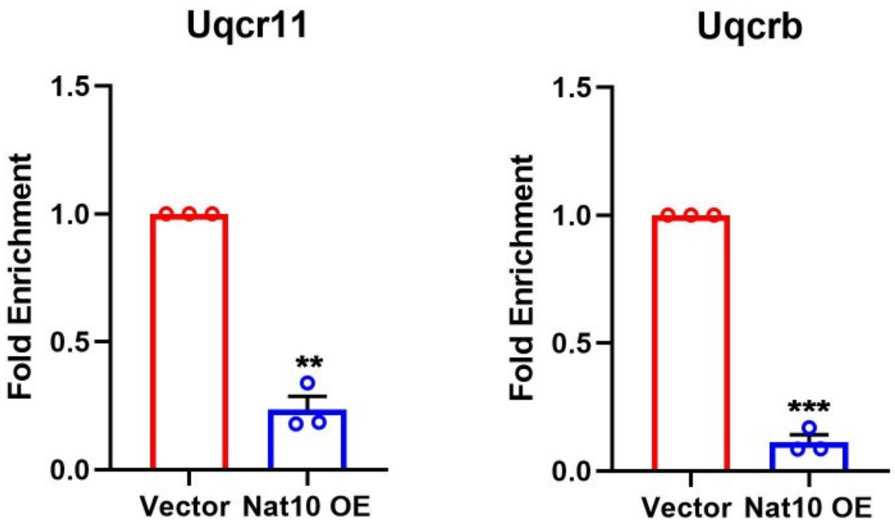

Supplementary Figure 8. The interaction of Uqcr11 and Uqcrb mRNAs with the Nuclear RNA Export Factor 1 (Nxf1). Nxf1 RIP analysis of Uqcr11 and Uqcrb (n=3). Two-tailed Student's t test with Welch's correction. $^{**}p < 0.01$, $^{***}p < 0.001$ vs. Vector. Data are presented as mean ± SEM. Source data are provided as a Source Data file.

Supplementary Fig. 9

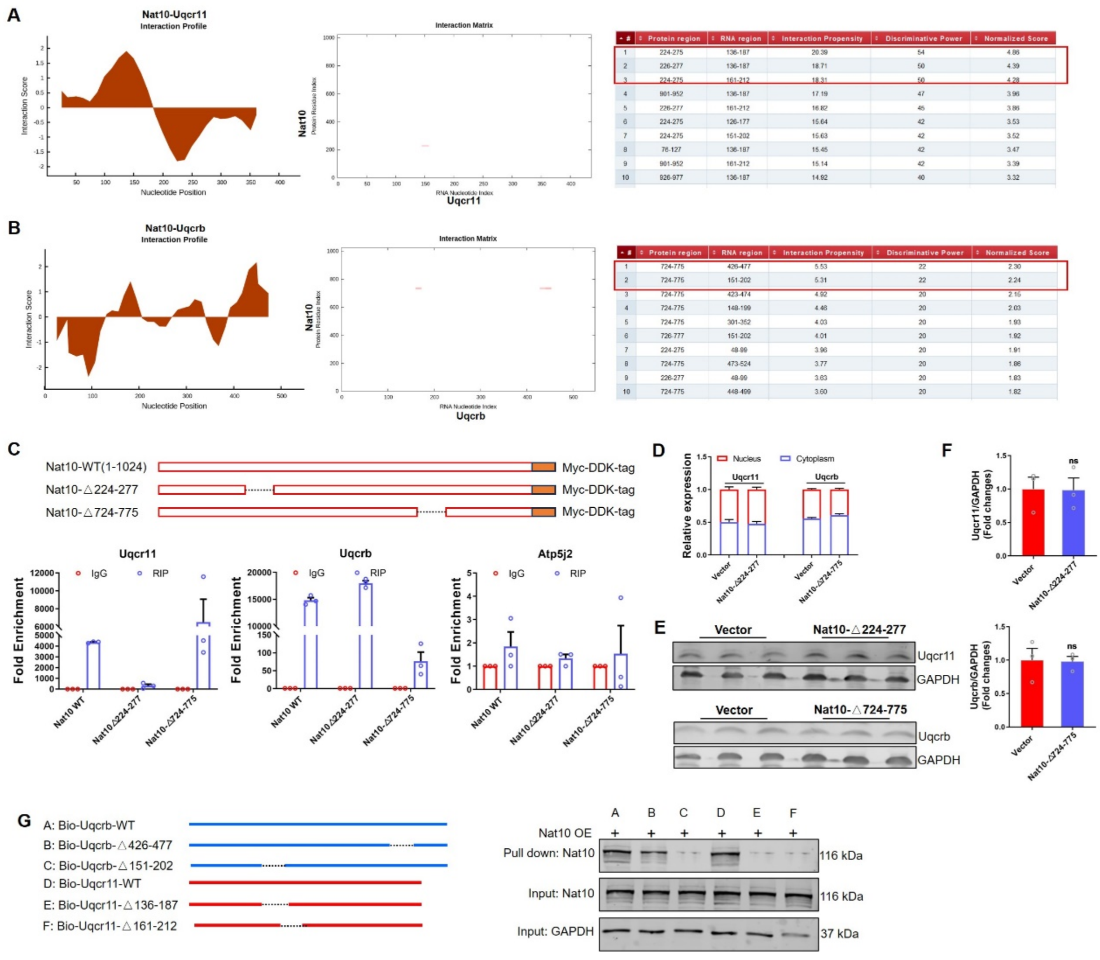

Supplementary Figure 9. The identification of the specific interection of Nat10 with Uqcr11 and Uqcrb. A and **B** The prediction of the binding sites between Nat10 and Uqcr11 or Uqcrb using the catRAPID algorithm. **C** The interaction of mutant Nat10 with Uqcr11 and uqcrb mRNA (n=3). **D** The distribution of Uqcr11 and Uqcrb between the nucleus and cytoplasm (n=3). **E** and **F** Western blot analysis of Uqcrb and Uqcr11 (n=3). **G** The interaction of mutant Uqcr11 and Uqcrb mRNAs with Nat10 protein. Two-tailed Student’s t test. Data are presented as mean ± SEM. Source data are provided as a Source Data file.

Supplementary Fig. 10

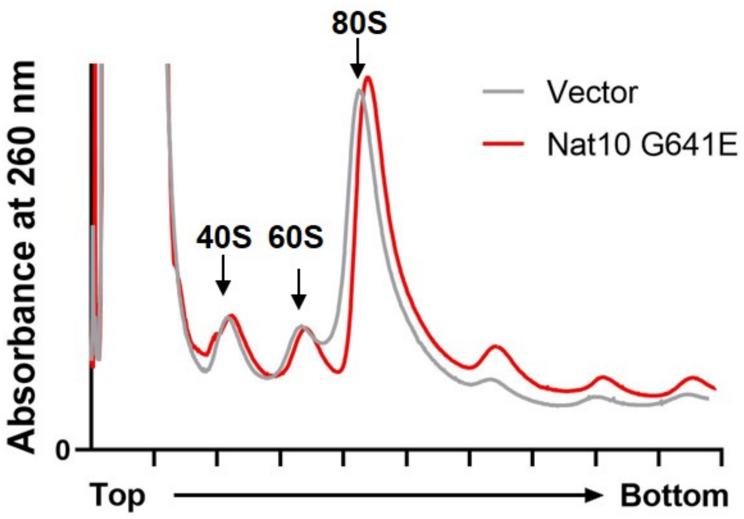

Supplementary Figure 10. The A254 absorbance data of Polysome profiling analyses. Source data are provided as a Source Data file.

Supplementary Fig. 11

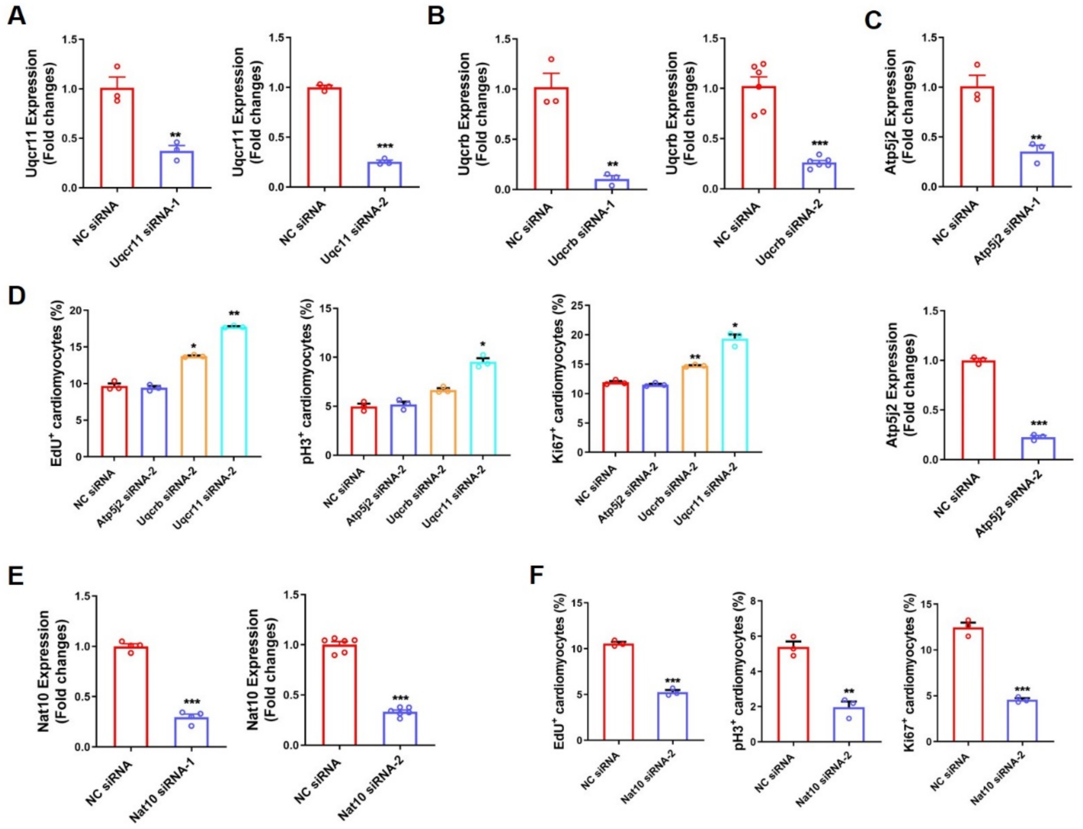

Supplementary Figure 11. **The effects of Nat10, Uqcrb, Uqcr11 and Atp5j2 siRNA on cardiomyocyte proliferation. A-C** and **E** the transfection efficiency of Nat10, Uqcrb, Uqcr11 and Atp5j2 siRNAs (n=3, 4, 6). Two-tailed Student's t test. **$p < 0.01$, ***$p < 0.001$ vs. NC siRNA. **D** and **F** The effects of Nat10, Uqcrb, Uqcr11 and Atp5j2 siRNA on cardiomyocyte proliferation analyzed by the detection of EdU, pH3 and Ki67 (n=3). one-way ANOVA followed by Dunnett's Multiple Comparison tests (D), Two-tailed Student's t test (F). *$p<0.05$, **$p < 0.01$, ***$p < 0.001$ vs. NC siRNA. Data are presented as mean ± SEM. Source data are provided as a Source Data file.

Supplementary Fig. 12

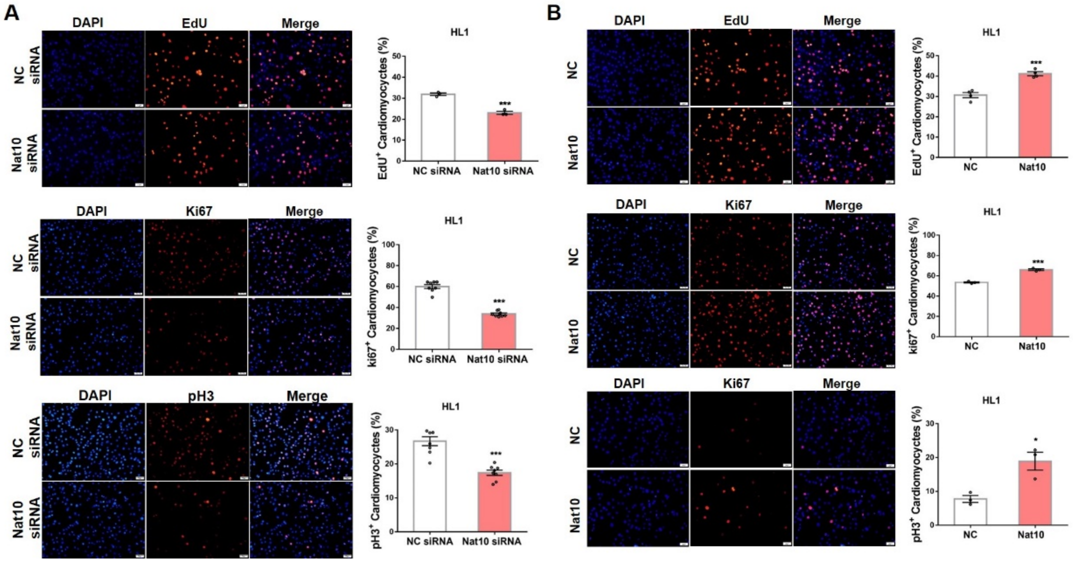

Supplementary Figure 12. The effects of Nat10 on HL1 cell proliferation. A and **B** The effects of Nat10 knockdown and overexpression on HL1 proliferation analyzed by the detection of EdU, pH3 and Ki67 (n=3, 8). Two-tailed Student’s t test. **p*<0.05, ****p* < 0.001 vs. NC siRNA or NC. Data are presented as mean ± SEM. Source data are provided as a Source Data file.

Supplementary Fig. 13

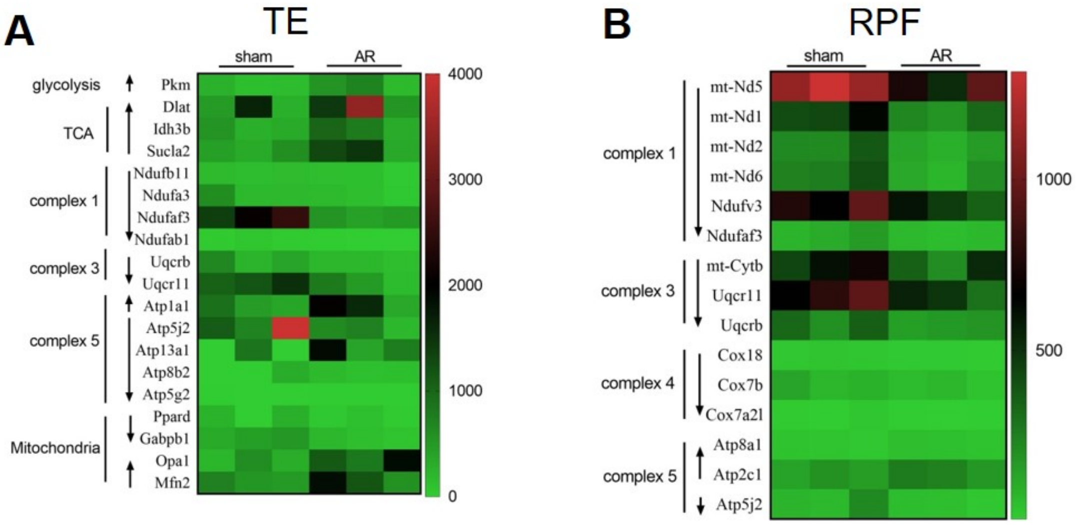

Supplementary Figure 13. Oxidative phosphorylation related genes in Ribo-seq. A and **B** Heatmaps of differentially expressed RPFs and TE of mitochondrial complex components related to oxidative phosphorylation and glycolysis-related gene in Ribo-seq. Source data are provided as a Source Data file.

Supplementary Fig. 14

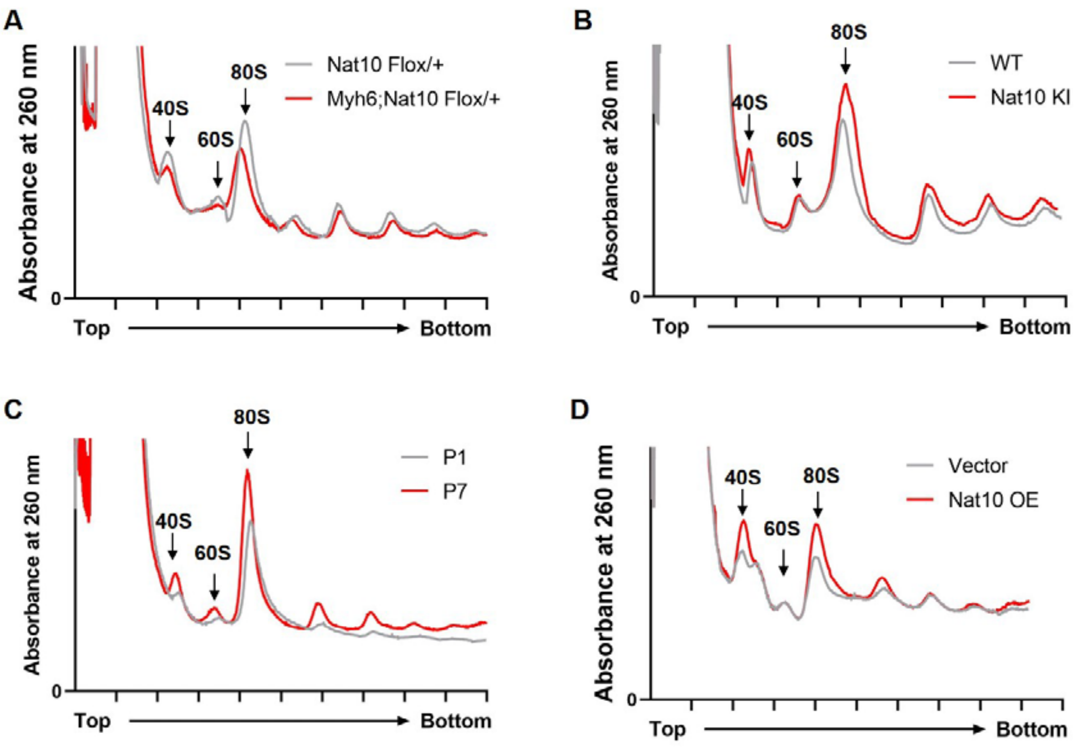

Supplementary Figure 14. **A-D** The A254 absorbance data of Polysome profiling analyses. Source data are provided as a Source Data file.

Supplementary Fig. 15

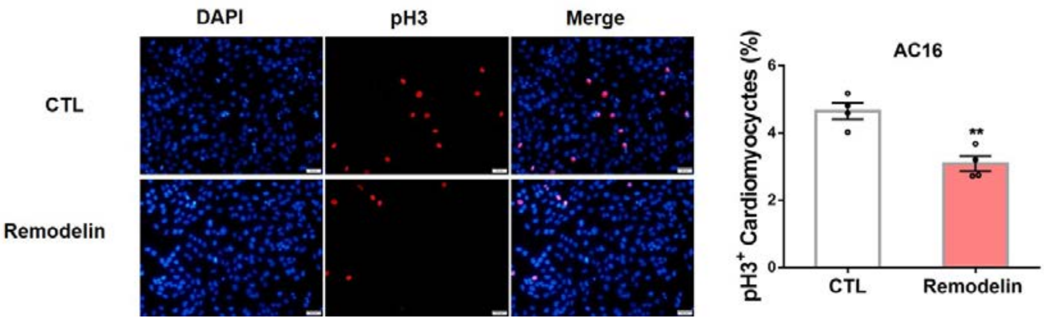

Supplementary Figure 15. The effects of Remodelin on AC16 cell proliferation. pH3 staining. **p < 0.01 vs. CTL. Two-tailed Student's t test. Data are presented as mean ± SEM.Source data are provided as a Source Data file.

Supplementary Fig. 16

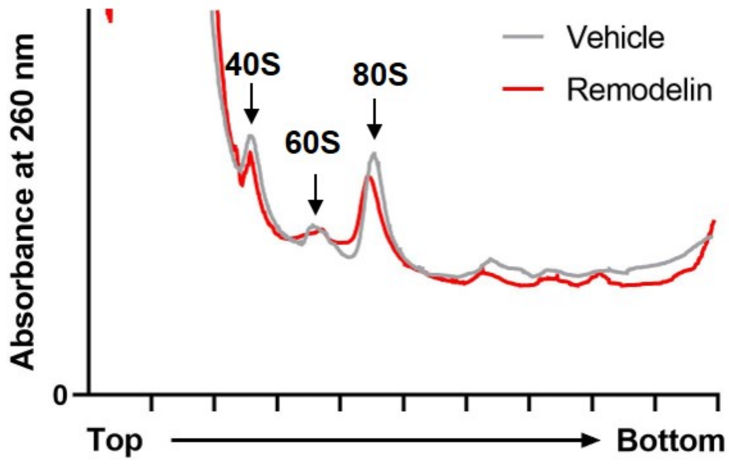

Supplementary Figure 16. The A254 absorbance data of Polysome profiling analyses. Source data are provided as a Source Data file.

Supplementary Fig. 17

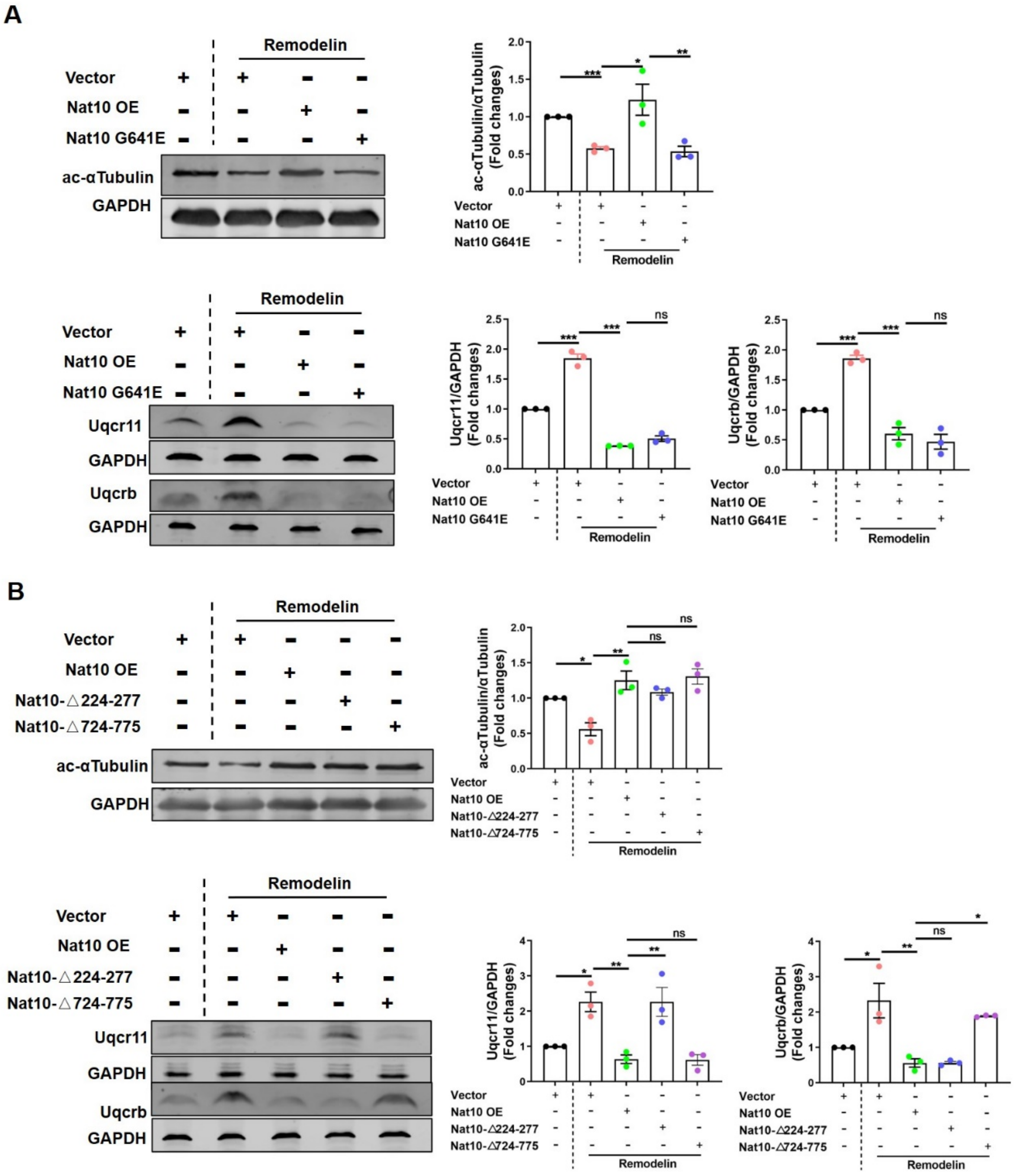

Supplementary Figure 17. **A** and **B** The effect of Remodelin on the expression of ac-α-tubulin, Uqcr11 and Uqcrb. **p* < 0.05, ***p* < 0.01, ****p* < 0.001. Data are presented as mean ± SEM. Source data are provided as a Source Data file.

Supplementary Fig. 18

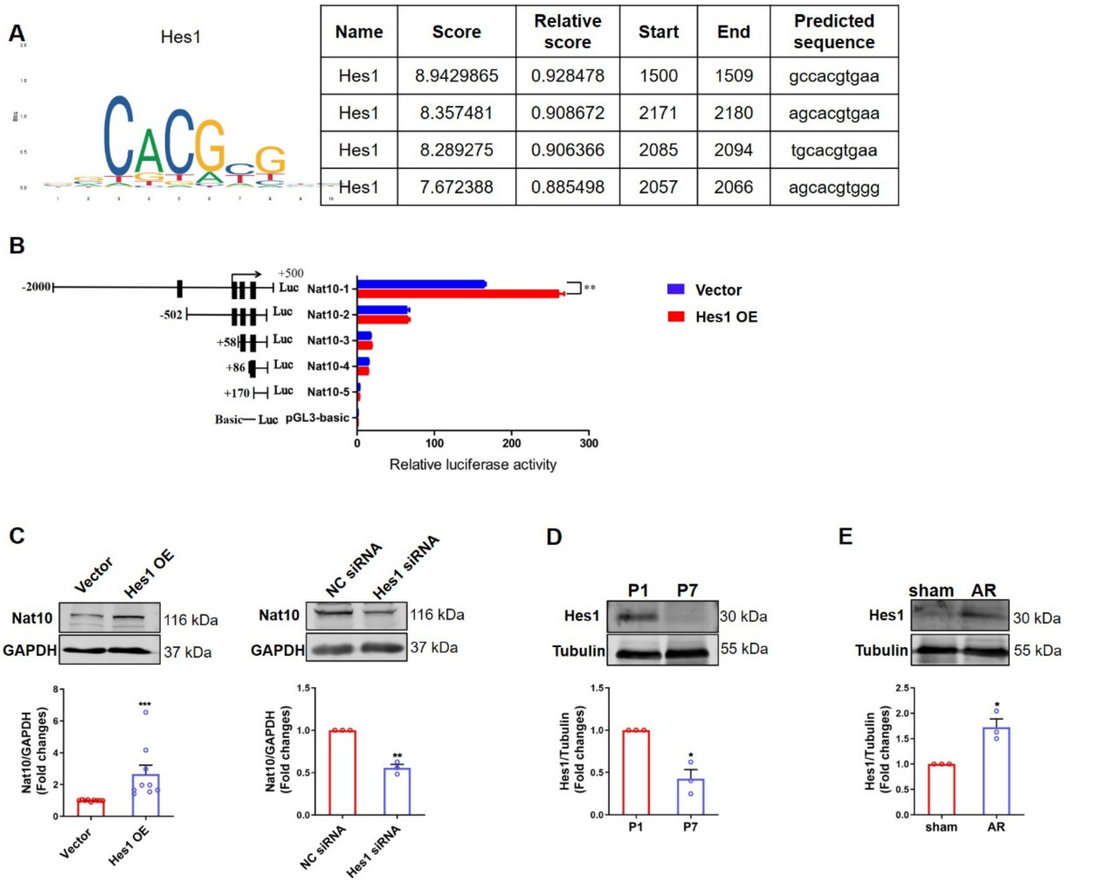

Name	Score	Relative score	Start	End	Predicted sequence
Hes1	8.9429865	0.928478	1500	1509	gccacgtgaa
Hes1	8.357481	0.908672	2171	2180	agcacgtgaa
Hes1	8.289275	0.906366	2085	2094	tgcacgtgaa
Hes1	7.672388	0.885498	2057	2066	agcacgtggg

Supplementary Figure 18. The expression of Nat10 is regulated by Hes1. A The prediction of Hes1 binding to the promoter region of Nat10 by both PROMO (https://alggen.lsi.upc.es/cgi-bin/promo_v3/promo/promoinit.cgi?dirDB=TF_8.3) and JASPAR (http://jaspar.genereg.net/) coupled with UCSC Genome Browser (http://genome.ucsc.edu). **B** The luciferase assay indicating the interaction of Hes1 with the promoter of Nat10. **C** The effects of Hes1 knockdown and overexpression on Nat10 protein expression. **D** The expression of Nat10 in P1 and P7 heart tissues. **E** The expression of Nat10 in hearts of sham and AR mice. Two-tailed Student's t test (C, D, and E). *$p < 0.05$, **$p < 0.01$, ***$p < 0.001$. Data are presented as mean ± SEM. Source data are provided as a Source Data file.
