# Supplementary material for: N-Acetyltransferase 10 represses Uqcr11 and Uqcrb independently of ac4C modification to promote heart regeneration
Source: Nat Commun. 2024 Mar 8;15:2137. doi: 10.1038/s41467-024-46458-7 (PMC10923914; doi:10.1038/s41467-024-46458-7)
Supplement: Supplementary file 3 — Reporting Summary [file 41467_2024_46458_MOESM3_ESM.pdf]

nature portfolio

Corresponding author(s): Benzhi Cai
Last updated by author(s): Jan 21, 2024

Reporting Summary

Nature Portfolio wishes to improve the reproducibility of the work that we publish. This form provides structure for consistency and transparency in reporting. For further information on Nature Portfolio policies, see our Editorial Policies and the Editorial Policy Checklist.

Statistics

For all statistical analyses, confirm that the following items are present in the figure legend, table legend, main text, or Methods section.

n/a	Confirmed	
☐	☒	The exact sample size (*n*) for each experimental group/condition, given as a discrete number and unit of measurement
☐	☒	A statement on whether measurements were taken from distinct samples or whether the same sample was measured repeatedly
☐	☒	The statistical test(s) used AND whether they are one- or two-sided<br>*Only common tests should be described solely by name; describe more complex techniques in the Methods section.*
☒	☐	A description of all covariates tested
☐	☒	A description of any assumptions or corrections, such as tests of normality and adjustment for multiple comparisons
☐	☒	A full description of the statistical parameters including central tendency (e.g. means) or other basic estimates (e.g. regression coefficient) AND variation (e.g. standard deviation) or associated estimates of uncertainty (e.g. confidence intervals)
☐	☒	For null hypothesis testing, the test statistic (e.g. *F*, *t*, *r*) with confidence intervals, effect sizes, degrees of freedom and *P* value noted<br>*Give P values as exact values whenever suitable.*
☒	☐	For Bayesian analysis, information on the choice of priors and Markov chain Monte Carlo settings
☒	☐	For hierarchical and complex designs, identification of the appropriate level for tests and full reporting of outcomes
☒	☐	Estimates of effect sizes (e.g. Cohen's *d*, Pearson's *r*), indicating how they were calculated

Our web collection on statistics for biologists contains articles on many of the points above.

Software and code

Policy information about availability of computer code

Data collection	Solexa pipeline version 1.8 (Off-Line Base Caller software, version 1.8) software, FastQC software, cutadapt, Bowtie software, STAR software, RiboTish software, Plastid software, edgeR package of R software, and RiboDiff software were used for sequencing data. Images were captured using confocal laser scanning microscope (FV300; Olympus, Japan).
Data analysis	Solexa pipeline version 1.8 (Off-Line Base Caller software, version 1.8) software, FastQC software, cutadapt, Bowtie software, STAR software, RiboTish software, Plastid software, edgeR package of R software, and RiboDiff software were used to generate and analyze sequencing data. KEGG analysis of changed genes by DAVID (https://david.ncifcrf.gov/home.jsp) and HIPLOT (https://hiplot.com.cn/cloud-tool/drawing-tool/list). A protein–protein interaction (PPI) network was constructed using STRING and visualized with Cytoscape (v. 3.7.1). Venn diagram was analyzed by HIPLOT. Images were analysed with ImageJ software. Statistical analyses were performed with GraphPad Prism 7.0.

For manuscripts utilizing custom algorithms or software that are central to the research but not yet described in published literature, software must be made available to editors and reviewers. We strongly encourage code deposition in a community repository (e.g. GitHub). See the Nature Portfolio guidelines for submitting code & software for further information.

Data

Policy information about availability of data

All manuscripts must include a data availability statement. This statement should provide the following information, where applicable:

- Accession codes, unique identifiers, or web links for publicly available datasets
- A description of any restrictions on data availability
- For clinical datasets or third party data, please ensure that the statement adheres to our policy

The sequencing raw data generated in this study have been deposited in Sequence Read Archive (SRA) at the NCBI Center with the accession number PRJNA974152 (https://www.ncbi.nlm.nih.gov/bioproject/PRJNA974152). All other data generated in this study are provided in the Source data file. The Source data are provided with this paper.

Research involving human participants, their data, or biological material

Policy information about studies with human participants or human data. See also policy information about sex, gender (identity/presentation), and sexual orientation and race, ethnicity and racism.

Reporting on sex and gender	Sex and gender were not considered in our study design
Reporting on race, ethnicity, or other socially relevant groupings	N/A
Population characteristics	The heart tissues we used are from 3 female human heart failing and 3 female non-heart failing patients.
Recruitment	Samples from 3 heart failure and non-heart failure patients were included in this study.
Ethics oversight	the Ethics Committee of Harbin Medical University

Note that full information on the approval of the study protocol must also be provided in the manuscript.

Field-specific reporting

Please select the one below that is the best fit for your research. If you are not sure, read the appropriate sections before making your selection.

[x] Life sciences   [ ] Behavioural & social sciences   [ ] Ecological, evolutionary & environmental sciences

For a reference copy of the document with all sections, see nature.com/documents/nr-reporting-summary-flat.pdf

Life sciences study design

All studies must disclose on these points even when the disclosure is negative.

Sample size	No sample size calculations were performed. The sample sizes were determined based on previous literature (Sadek HA et al. 2013, Nature), as well as allowing for statistical analyses such as calculation of standard deviation and performing t-tests. In vitro studies were repeated a minimum of three times for independence and the in vivo sample sizes were determined following established standards for animal studies, with a minimum of n=3 independent experiments for adequate reproducibility.
Data exclusions	No data were excluded.
Replication	The data obtained in our study were from at least 3 independently experiments. Details are described in the legends of the corresponding figures.
Randomization	All samples were randomly allocated into experimental groups.
Blinding	We analyzed samples as blindly as possible by performing samples treatment, data collection, and analyses through different investigators.

Reporting for specific materials, systems and methods

We require information from authors about some types of materials, experimental systems and methods used in many studies. Here, indicate whether each material, system or method listed is relevant to your study. If you are not sure if a list item applies to your research, read the appropriate section before selecting a response.

Materials & experimental systems

n/a	Involved in the study
	[x] Antibodies
	[x] Eukaryotic cell lines
[x]	Palaeontology and archaeology
	[x] Animals and other organisms
[x]	Clinical data
[x]	Dual use research of concern
[x]	Plants

Methods

n/a	Involved in the study
[x]	ChIP-seq
[x]	Flow cytometry
[x]	MRI-based neuroimaging

Antibodies

Antibodies used

α-actinin (Genetex, GTX29465, 1:400 for IF)
phosphoHi-stone H3 (pH3; Millipore, #06–570, 1:400 for IF)
Ki67(Abcam, ab15580, 1:400 for IF)
Uqcrb (Proteintech, 10756-1-AP, 1:1000 for WB)
Uqcr11 (Immunoway, YN4606, 1:1000 for WB)
ATP5j2 (Proteintech, 68128-1-Ig, 1:1000 for WB)
Nat10 (Proteintech, 13365-1-AP, 1:1000 for WB, 5 μg for RIP)
ac4C (Abcam, ab252215, 1:500 for dot blot)
Hes1(Abways, CY5649, 1:500 for WB)
GAPDH (Abways, AB0037, 1:1000 for WB)
Tubulin (Absin, abs830032, 1:1000 for WB)
Alexa Fluor 488 (Abcam, ab150113, 1:400 for IF)
Alexa Fluor 594 (Abcam, ab150080 1:400 for IF)

Validation

All antibodies used in this study were obtained from commercial sources and were validated in multiple previous studies. Validation information for each of the antibodies used in the study can be found on the manufacturer's website.
α-actinin: from mouse; Species reactivity: Human, Mouse, Zebrafish; application: ICC/IF, IHC-P, IHC-Fr, IHC-Wm.
pH3: from rabbit; Species reactivity: mouse, human; application: ICC/IF, IP, WB.
Ki67: from rabbit; Species reactivity: mouse, human; application: IHC-P, ICC/IF.
Uqcrb: from rabbit; Species reactivity: mouse, human, rat; application: WB, IP, IHC, ELISA.
Uqcr11: from rabbit; Species reactivity: mouse, human; application: WB.
Atp5j2: from mouse; Species reactivity: Human, Mouse, Rat, Rabbit; application: WB, IF, ELISA.
Nat10: from rabbit; Species reactivity: mouse, human, rat; application: WB, RIP, IP, IHC, IF, CoIP, ELISA.
ac4C: from rabbit; Species reactivity: mouse; Application: dot blot.
Hes1: from rabbit; Species reactivity: mouse, human, rat; application: WB, IHC, ICC/IF, FC.
GAPDH: from rabbit; Species reactivity: Human, Mouse, Rat, Mk, Ch, Ze, Fish; Application: WB, IHC, ICC/IF, IP, FC.
Tubulin: from mouse; Species reactivity: Human, Mouse, African green monkey; Application: WB.

Eukaryotic cell lines

Policy information about cell lines and Sex and Gender in Research

Cell line source(s)

AC16 (human, ATCC, BFN60808678) and HL-1 (mouse, ATCC, BFN60808678) were from BLUEFBIO life Science. The hESCs were obtained from the National Stem Cell Resource Center.

Authentication

The cell lines were confirmed through short tandem repeat DNA profiling used within six months for testing. All cell lines were kept at low passages in order to maintain their identity.

Mycoplasma contamination

All cell lines tested were negative for mycoplasma contamination.

Commonly misidentified lines
(See ICLAC register)

No commonly misidentified cell lines were used in the study.

Animals and other research organisms

Policy information about studies involving animals; ARRIVE guidelines recommended for reporting animal research, and Sex and Gender in Research

Laboratory animals

The neonatal mice aged 1-2 days and male/female C57BL/6 mice aged 8 - 10 weeks were purchased from the Laboratory Animal Center of the Second Affiliated Hospital of Harbin Medical University (Harbin, China). The Nat10 f/f, Nat10 KI, and Myh6-Cre mice on C57BL/6 background were purchased from Cyagen, China. The apex resection (AR) was performed in P1 knockout mice and myocardial infarction experiments were performed in adult (8 - 10 weeks) knockin male mice. The postnatal day 1 (P1) and day 7 (P7) bama miniature pigs were purchased from Harbin Veterinary Research Institute, Chinese Academy of Agricultural Sciences. The

	hearts of P1 and P7 pigs, which did not undergo any damage or experiments prior to harvesting, were harvested for analysis. Animals were housed under a temperature (20-24°C) and humidity (40–60%) with a 12-h light/dark cycle. They were provided with unrestricted access to food and water.
Wild animals	The study did not involve wild animals.
Reporting on sex	There was no sex bias in the animals used in this study due to the use of neonatal mice.
Field-collected samples	The study did not involve samples collected from the field.
Ethics oversight	The animal studies were approved by the Animal Care and Use Committee of Harbin Medical University. All procedures complies with the Guide for the Care and Use of Laboratory Animals published by the National Institutes of Health.

Note that full information on the approval of the study protocol must also be provided in the manuscript.
